# A combined treatment regimen for *Trichuris rhinopiptheroxella* infection in *Rhinopithecus roxellana* in southern China

**DOI:** 10.1016/j.ijppaw.2025.101036

**Published:** 2025-01-03

**Authors:** Zhengjiu Ren, Jinzhi Lu, Tianyou Zhang, Lihua Xiao, Peng Zhang, Guixin Dong, Yaoyu Feng, Dongjuan Yuan

**Affiliations:** a State Key Laboratory for Animal Disease Control and Prevention, Center for Emerging and Zoonotic Diseases, College of Veterinary Medicine, South China Agricultural University, Guangzhou, Guangdong, 510642, China; b Guangdong South China Rare Wild Animal Species Conservation Center, Zhuhai, 519031, China; c Chimelong Group Co., Guangdong, 511430, China

**Keywords:** *Rhinopithecus roxellana*, *Trichuris rhinopiptheroxella*, Regimen, Administration, Control

## Abstract

The Sichuan snub-nosed monkey (*Rhinopithecus roxellana*) is an endangered species in China and is susceptible to infection with *Trichuris* species. However, the worms are difficult to remove completely. A practical treatment regimen for trichuriasis was conducted over a seven-month period on 15 *R. roxellana* in a wildlife zoo in southern China. Initially, a combination of fecal examination, morphological observation, molecular identification of ITS1 and mitogenome, and infective pattern analysis revealed that *R. roxellana* was susceptible to *Trichuris rhinopiptheroxella*. Three rounds of treatment were administrated, with a dosage of 10 mg/kg albendazole or ivermectin in each. The initial administration of albendazole to 15 monkeys resulted in a reduction of fecal eggs per gram (EPG) by 7.8%–73.2%. The subsequent administration of albendazole to 9 monkeys demonstrated a reduction in fecal EPG by 52.6%–52.8%. The third administration of ivermectin to 5 monkeys resulted in a reduction of fecal EPG by 55.6%–96.6%. However, the EPG level increased in some monkeys after one month of these three anthelmintic administrations. Subsequently, improved strategies were implemented, including an increased dosage of albendazole, flame sterilization, the replacement of the sandy floor with concrete, and the hanging of food. Results showed that a reduction in the fecal EPG of 8 monkeys in the exhibition region, with a decrease from 5135 to 63. Additionally, 6 monkeys exhibited a negative EPG after one month. In the breeding region, the fecal EPG of 7 monkeys decreased from 7389 to 869. Additionally, 2 monkeys demonstrated a negative EPG after one month. This study provides evidence for the control of *Trichuris* infection in *R. roxellana* and offers a guideline for the treatment of trichuriasis in animals.

## Highlights

• *Rhinopithecus roxellana* is susceptible to *Trichuris rhinopiptheroxella.*
• Anthelmintic treatment has the limited effects on trichuriasis of *R. roxellana* due to reinfection of *T. rhinopiptheroxella.*
• Implementation of comprehensive management strategies has the potential to the treatment of trichuriasis in *R. roxellana*.

## Graphical abstract

**Figure:**
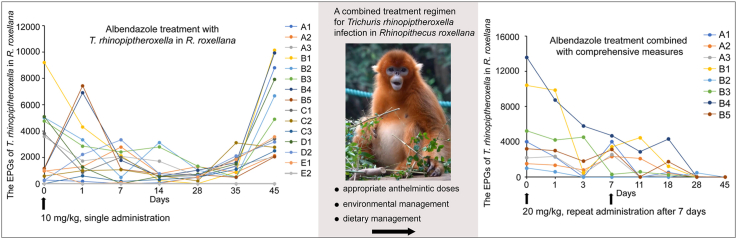

## 1 Introduction

The Sichuan snub-nosed monkey (*Rhinopithecus roxellana*) has been classified as a Class I protected species by the national government of China and the Convention on International Trade in Endangered Species of Wild Fauna and Flora (CITES). Furthermore, the species is classified as vulnerable on the International Union for Conservation of Nature (IUCN) Red List of Threatened Species (Fang et al., 2018; Luo et al., 2012). The species is primarily distributed in southwestern Gansu Province, the Qinling Mountains in Shaanxi Province, the Shennongjia Forestry District in Hubei Province, and Sichuan Province in China (Luo et al., 2012). As one of the China's endemic and endangered species, *R. roxellana* has been residing in the Wildlife Zoo and Nature Reserves Center of China (Liu et al., 2024).

*Trichuris* species parasitizes the intestine of *R. roxellana,* resulting in a range of adverse effects, including anemia, typhlitis, colitis, chronic dysentery, severe malnutrition, and even death (Khuroo et al., 2010; Wang et al., 2019). Our previous study demonstrated that *Trichuris* species was the predominant nematode observed in arboreal primates of golden monkeys and white-cheeked gibbons, as well as in terrestrial primates of baboons and patas monkeys, in several wildlife zoos in southern China (Li et al., 2024). Other studies demonstrated that the prevalence of *Trichuris* in fecal samples of *R. roxellana* from the zoos in China was 80.0%, with positive samples exhibiting an egg per gram (EPG) of 33,275 ± 6557 for *Trichuris* species (Li et al., 2015). The prevalence of *Trichuris* species in *R. roxellana* was high in the zoos, which poses a threat to the breeding and conservation of *R. roxellana* (Wang et al., 2019).

The fecal eggs of *Trichuris* species have the potential to develop into infective eggs within a period of approximately one to two months under conditions of optimal temperature (22–28 °C), humidity, and sufficient oxygen. Infective eggs are ingested by the susceptible animals via fecal-oral transmission and are decapsulated into L1 larvae. Subsequently, larva penetrates the cecum and proximal colonic wall, where it resides within the epithelium. L1 larvae develop into L2 larvae at 9–11 days post-infection (dpi), L3 larvae at 17 dpi, and L4 larvae at 22 dpi. Female worm is capable of ovulating approximately 3000 to 7000 eggs per day during the peak period. Adult worms can survive within the host intestine for up to 3–5 years. Infected animals serve as the source of transmission for *Trichuris* species (Klementowicz et al., 2012). As a soil-transmitted helminth, conventional methods for identifying *Trichuris* species are microscopic examination of fecal eggs and adult worms and molecular approaches (Nikolay et al., 2014).

Mebendazole, albendazole, and ivermectin are commonly utilized drugs for treatment of trichuriasis. The prophylactic treatment of *R. roxellana* in zoo entails the implementation of regular fecal egg detection and the utilization of albendazole and mebendazole to prevent *Trichuris* infection in susceptible populations (Anderson et al., 2012). However, fecal EPGs of *R*. *roxellana* increased after one month, and the efficacy of anthelmintic drugs for trichuriasis treatment was limited (He et al., 2017). In this study, we employed a dual approach, combining microscopic examination and molecular analysis of fecal worms, to identify the worm present in *R. roxellana* at one wildlife zoo in southern China. Then, a practical treatment regimen for trichuriasis was conducted over a seven-month period. The objective of this study was to develop an effective treatment for trichuriasis in captive *R. roxellana*. The study yielded evidence that can inform the prevention and control of *Trichuris* infection in *R. roxellana*.

## 2 Material and methods

### 2.1 Ethics statement

All fecal samples utilized in this study were obtained from *R. roxellana* with the approval of the zoo. All experimental procedures were conducted in accordance with the Animal Ethical Procedures and Guidelines of the People's Republic of China. The research protocol was reviewed and approved by the Ethics Committee of South China Agricultural University, People's Republic of China.

### 2.2 The *R. roxellana* family in the wildlife zoo

**Fig. 1:**
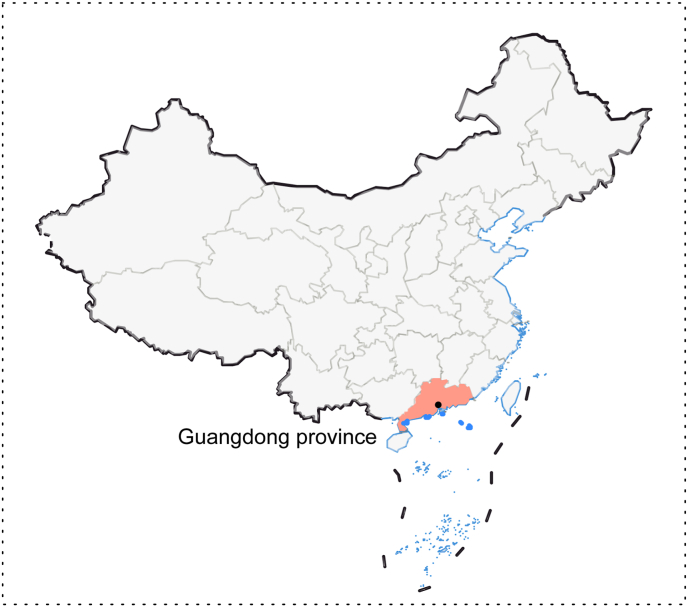
**Map of the Wildlife Zoo in Guangdong Province of the southern China.** Red regions were the map of Guangdong province. Dark circle was the sample site.

A total of fifteen *R. roxellana* were captive at the Chimelong wildlife zoo in Guangdong Province, southern China (Fig. 1). The members of the *R. roxellana* family were 15 and were listed in Table 1. The mesh cage for *Rhinopithecus roxellana* has 1 m above the ground, and the feces of monkeys can fall onto the ground through the mesh gaps. The breeder cleans the cage and the ground with about 70 °C hot water every day. The activity area in the exhibition region is the cement ground, while the activity area in the breeding region is the sandy ground. The main food sources for these monkeys are the leaves of mulberry, holly, and redbud. The fresh leaves are supplied to these monkeys in the morning and evening, respectively. The leaves were placed on the table in the activity area in the morning, and the leaves were tied on the cage in the evening. Veterinary physician conducts deworming treatment on the monkeys twice a quarter by rotating the usage of albendazole and benzimidazole. The conventional dose is a single dose of 10 mg/kg, but occasionally individual deworming treatment is performed on the monkeys with the high EPG level.

**Table 1:** The status of the *R. roxellana* family members.

Symbol		Gender	Age (year, growth stage)	Body weight (kg)	Living region
A	A1	M	12 (adult)	15.8	Exhibition region
	A2	F	15 (adult)	11.0	
	A3	F	14 (adult)	11.2	
B	B1	M	6 (subadult)	8.6	
	B2	M	5 (juvenile)	10.8	
	B3	M	8 (adult)	19.2	
	B4	M	7 (subadult)	12.0	
	B5	M	9 (adult)	16.6	
C	C1	F	9 (adult)	7.2	Breeding region
	C2	M	2 (childhood)	3.4	
	C3	M	12 (adult)	17.0	
D	D1	M	1 (childhood)	1.8	
	D2	F	7 (subadult)	8.6	
E	E1	M	11 (adult)	15.6	
	E2	F	10 (adult)	8.6	

### 2.3 Feces collection and eggs and adult worms’ observation

The feces were collected and identified at the individual animal level. The fecal samples from 15 *R. roxellana* were collected once at approximately 2 weeks interval in previous 6-month study. The fecal samples from 15 *R. roxellana* were collected once at approximately 3–4 days interval within 15 days after a series of combined measures were implemented. Then, the fecal samples from 15 *R. roxellana* were collected once at 7, 10, and 13 days intervals, respectively, because of the low EPG level in fecal samples. Thus, a total of 384 fecal samples were collected from 15 *R. roxellana* between June 2023 and December 2023. The number of average fecal samples in each *R. roxellana* was approximately 25.

Fresh feces were obtained from each *R. roxellana* and stored at 4 °C. Subsequently, approximately 400 mg fecal samples were soaked in 4 mL saturated salt buffer and thoroughly mixed by vortexing. Fecal suspension was moved to McMaster egg counting chamber to calculate the average number of floating eggs present in the buffer after 5 min stewing. The EPG was calculated by this formula: Y = 4/0.15∗X/M, where X: the number of eggs in McMaster egg counting chamber, M: the mass of fecal sample in 4 mL EP tube. The fecal samples with the high EPG level were soaked and washed by phosphate-buffered saline (PBS) to remove plant residue. Fecal sample was filtered by double layer gauze several times and the sediments were used to isolate adult worms under stereomicroscope. Only three adult worms were found in one *R. roxellana* fecal sample. Subsequently, the worms were immediately frozen in formalin. A microscopic examination was conducted to observe the morphology of the eggs and adult worms using Leica S9i Stereo microscope (Leica, Wetzlar, Germany).

### 2.4 Fecal DNA extraction and PCR amplification of *trichuris* internal transcribed spacer 1 (ITS1)

Approximately 200 mg of monkey feces were subjected to wash three times with PBS buffer, followed by centrifugation at 2000×*g* for 10 min. Genomic DNA was extracted from the fecal samples using the Fast DNA SPIN Kit for Soil (MP Biomedicals, CA, USA), and extracted DNA was subsequently stored at −20 °C. PCR primers for ITS1 gene were employed to detect *Trichuris* species in the DNA of fecal samples (Table S1) (Ghai et al., 2014). The first round of PCR for ITS1 gene (∼1080 bp) was conducted as follows: 94 °C for 1 min denaturation; 40 cycles at 94 °C for 1 min, 61 °C for 30 s, and 72 °C for 75 s; followed by a final extension at 72 °C for 10 min. The nested PCR reaction for ITS1 gene (∼890 bp) was conducted as follows: 94 °C for 1 min denaturation; 35 cycles at 94 °C for 30 s, 55 °C for 30 s, and 72 °C for 75 s; followed by a final extension at 72 °C for 10 min. The nested round of PCR for ITS1 gene were sequenced on an ABI 3730 Genetic Analyzer (Applied Biosystems, CA, USA). The nucleotide sequences were assembled using ChromasPro v.1.32 (http://technelysium.com.au/ChromasPro.html) and aligned to GenBank reference sequences for nematode identification using ClustalX v.2.1 (http://clustal.org).

### 2.5 Eggs DNA extraction and mitogenome analysis

Worm eggs were collected from *R. roxellana* feces by saturated salt flotation method. The eggs were completely washed with PBS buffer, and total genomic DNA was extracted from eggs using DNeasy Blood & Tissue Kit (Qiagen, Hilden, Germany) in accordance with manufacturer's instructions and then stored at −20 °C until utilization. The total DNA was sequenced on an Illumina Novaseq 6000 sequencing platform using a PE150 technique (Personalbio Co.). Subsequently, quality control filtering was conducted using Fastp (v 0.23.2) on 46.6 Gb of raw data to remove low-quality reads and sequencing adapters, and approximately 44.2 Gb of data were obtained. Furthermore, the filtering of *R. roxellana* gene sequences (accession number: GCF_000769185.1) was conducted using bowtie2 (v 2.5.2) and SAMtools (v 1.3.1), and approximately 43.8 Gb of data were obtained. The assembly of worm mitogenome was conducted using GetOrganlle (v 1.7.6.1) and Novoplasty (v 4.2). The complete mitogenome of the worm was annotated using Mitos (http://mitos.bioinf.uni-leipzig.de/index.py) and Geseq (https://chlorobox.mpimp-golm.mpg.de/geseq.html). The boundaries of protein-coding genes (PCGs) were determined by comparison with the mitogenomes of *Trichuris* spp. from the primates (accession number: MW448472, MG189593, KT449824, KC461179, NC_017750, and KT449825). The organization of mitogenome was visualized with MTviz (http://pacosy.informatik.uni-leipzig.de/mtviz/mtviz). The 13 PCGs of the worm and 23 *Trichuri*s species were aligned using MAFFT (v7.508) (Katoh et al., 2017), and a maximum likelihood phylogenetic tree was constructed using RAxML-ng at ModelTest-NG (v0.1.7) with GTR + I + G4 model (Darriba et al., 2019).

### 2.6 Statistical analysis

A multifactorial correlation analysis of the EPG levels with sex, age, habitat, and diarrhea was conducted using Mann-Whitney and Kruskal-Wallis tests, and *P* < 0.05 was considered statistically significant. Furthermore, a logistic regression analysis was conducted using a one-way regression analysis to evaluate the impact of fecal EPG levels on the incidence of diarrhea in *R. roxellana*, and *P* < 0.05 was considered statistically significant. Subsequently, an odds ratio (OR) and a 95% confidence interval (CI) were calculated to analyze the association between diarrhea and exposure factors of *R. roxellana*. At *P* < 0.05, if OR > 1, the factor may be a risk factor for diarrhea; if 0 < OR < 1, the factor may be a protective factor for diarrhea and could potentially cause diarrhea; and if OR = 1, the factor has not the risk factor for diarrhea. All data were analyzed using statistical software package SPSS 27.0 (SPSS Inc., Chicago, USA).

## 3 Results

### 3.1 The *R. roxellana* family

The 15 members of the *R. roxellana* family exhibited a range of ages, with a mean of 8.5 years and a standard deviation of 4.0 years. A total of 60.0% of the monkeys were adults, aged over 8 years. A further 20% were subadults, with an age of 6–7 years. The juvenile monkeys (aged 3–5 years) constituted 6.7%, while the remaining 13.3% were children, aged less than 2 years (Table 1). The body weight of 15 monkeys ranged from 1.8 to 19.2 kg (Table 1). Of 15 monkeys, 9 were male and 6 were female. Some of them demonstrated a relationship as shown in Fig. S1. Fifteen *R. roxellana* were divided into 5 groups based on whether they were kept in the same cage. Of 8 monkeys in the exhibition region, 3 were assigned to group A and 5 to group B. Seven monkeys in the breeding region were divided into 3 in group C, 2 in group D, and 2 in group E (Table 1 and Fig. S1).

### 3.2 Morphological and molecular identification of worms from *R. roxellana* feces

**Fig. 2:**
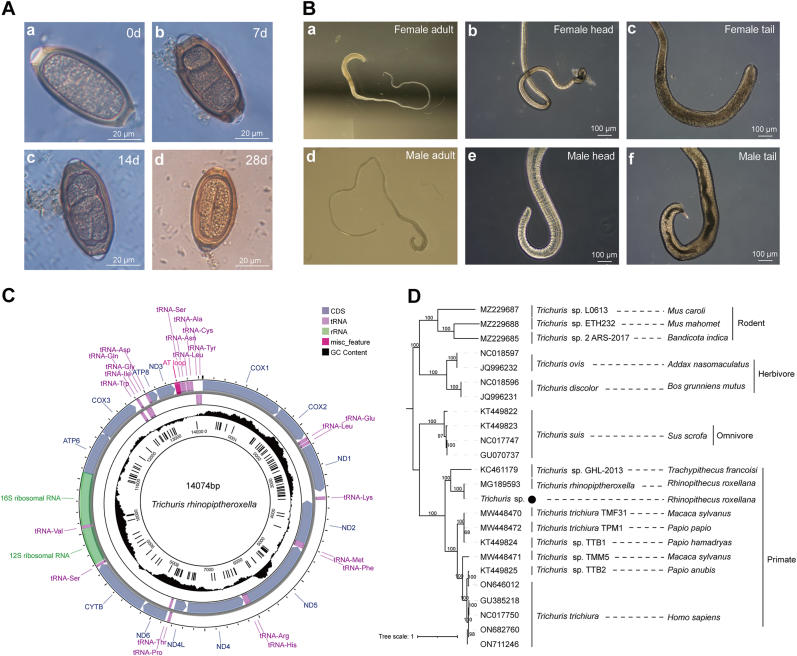
Morphology and mitogenome of *Trichuris* sp*.* from *R. roxellana.* (A) Microscopic examination of the egg hatching of *Trichuris* species at 0 day, 7 days, 14 days, and 28 days. (B) Microscopic examination of adult worms from the feces of *R. roxellana*. (C) Mitogenome organization of *Trichuris* species from *R. roxellana*. The map shows 13 PCGs, 21 transfer RNA, 2 rRNA, and 1 NCR. The two outer circles consist of 13 PCGs and their coding directions; The inner circle contains SNP loci. (D) Phylogenetic tree of mitogenome sequences of *Trichuris* species from animals. This tree was reconstructed using the maximum likelihood method. Bootstrap values were generated using 1000 replicates. Nodes with bootstrap values of 50 or greater were indicated. The scale bar indicated the number of nucleotide substitutions per site. The black round indicated the worm from *R. roxellana*.

Microscopic examination of the fecal samples of *R. roxellana* revealed the presence of brown, barrel-shaped eggs with thick eggshells and egg plugs at both ends. The eggs were 64.92 ± 9.46 μm in length and 33.04 ± 5.91 μm in width (Fig. 2A). Three adult worms isolated from the feces were observed to be white, round-shaped, with a slender anterior end and a thick and short posterior end, forming a typical whip-shape. The male worms exhibited a spicule at the terminus of the tail (Fig. 2B). The morphological characteristics of eggs and adult worms were consistent with those observed in *Trichuris* species. The one-celled eggs could develop into the eggs containing larvae on the 28th day of incubation at 28 °C, indicating that the eggs had the potential to develop into infective eggs (Fig. 2A).

The amplification of ITS1 gene from total DNA of worm eggs and adult worm was 890 bp, which was subsequently deposited in GenBank database (accession number: OR563921) (Figs. S2 and S3). The sequence demonstrated a high sequence identity of 99.88% with ITS1 gene of *Trichuris* sp. Rr1 GY-2019 from GenBank database (accession number: MN447324). A phylogenetic analysis showed that ITS1 sequences of *Trichuris* species from primates, herbivores, omnivores, and rodents were clustered into four branches, whereas *Trichuris* species in *R. roxellana* from the zoos in southern China were clustered into a single branch with ITS1 sequence of *Trichuris* sp. Rr1 GY-2019 (Fig. S4).

### 3.3 Mitogenome analysis of *T. rhinopiptheroxella*

A total of 46.6 Gb of raw data were obtained from the complete genomic DNA of the worm through Illumina sequencing. The assembled sequences revealed the worm mitogenome with a size of 14,074 bp using Getorganlle and Novoplasty software (Fig. 2C). The mitogenome sequence was deposited in GenBank with accession number PQ247221. The worm mitogenome had the highest sequence identity (99.2%) with *T. rhinopiptheroxella* mitogenome (accession number: MG189593) from *R. roxellana* in Shaanxi Province, northwestern China. The worm mitogenome was a circular DNA molecule comprising 37 genes, including 13 PCGs, 21 tRNA genes (two coding for leucine), 2 rRNA genes, and 1 non-coding region (NCR) (Fig. 2C). A comparison of the worm mitogenome with that of *T. rhinopiptheroxella* in *R. roxellana* from Shaanxi Province in northwestern China showed that four out of 13 PCGs (*nad*4L, *nad*6, *atp*6, and *atp*8) had different start codons, namely ATA, ATT, ATA, and ATT, respectively. These differeces in start codons of ACA, ATA, CTA and GAT were observed in *T. rhinopiptheroxella* in *R. roxellana* from Shaanxi Province (Table S2) (Wang et al., 2019). Two out of 13 PCGs (*nad*4L and *atp*8) had different stop codons (TAA and TAG) compared to stop codons (TAG and T) observed in *T. rhinopiptheroxella* in *R. roxellana* from Shaanxi Province (Table S2) (Wang et al., 2019). The length of worm *rrn*S was 10 bp shorter than that of *T. rhinopiptheroxella* in *R. roxellana* from Shaanxi Province, while *rrn*L was identical. A phylogenetic analysis was conducted on 13 PCGs present in the mitogenomes of 24 *Trichuris* species, representing a diverse range of hosts, including rodents, herbivores, omnivores, and primates. In the primate group, the worm was grouped in a single branch with *T. rhinopiptheroxella* from *R. roxellana* in Shaanxi Province (Fig. 2D). Additionally, both worms showed the similar morphological characteristics (Wang et al., 2019). Thus, the worm from *R. roxellana* can be identified as *T. rhinopiptheroxella* based on morphological observation, single-molecular identification, and mitogenome analysis.

### 3.4 Infection of *T. rhinopiptheroxella* and diarrhea of *R. roxellana*

A total of 106 fecal samples were collected from 15 monkeys through a 3-month study. Morphological observation and ITS1 identification of the fecal samples showed that overall infection rate of *T. rhinopiptheroxella* in 15 *R. roxellana* was 90.6% (96/106) (Fig. S5). Furthermore, the mean diarrhea rate of 90 fecal samples from 15 monkeys was 31.7%. The fecal samples were then classified into four grades in accordance with previous study (Yi et al., 2020). The association analyses indicated that there was no correlation between the EPG of *R. roxellana* (n = 15) and sex (*P* = 0.391), age (*P* = 0.245), cohabitation (*P* = 0.45), or living region (*P* = 0.728). However, a significant correlation was observed between the EPG of *R. roxellana* and diarrhea (*P* = 0.022) (Table S3). The logistic regression analysis also demonstrated a significant correlation between the EPG of *R. roxellana* and the occurrence of diarrhea (OR = 1.002, 95% CI = 1.001–1.002, *P* < 0.01) (Table S4).

### 3.5 The impacts of anthelmintic treatment on *T. rhinopiptheroxella* in *R. roxellana*

**Fig. 3:**
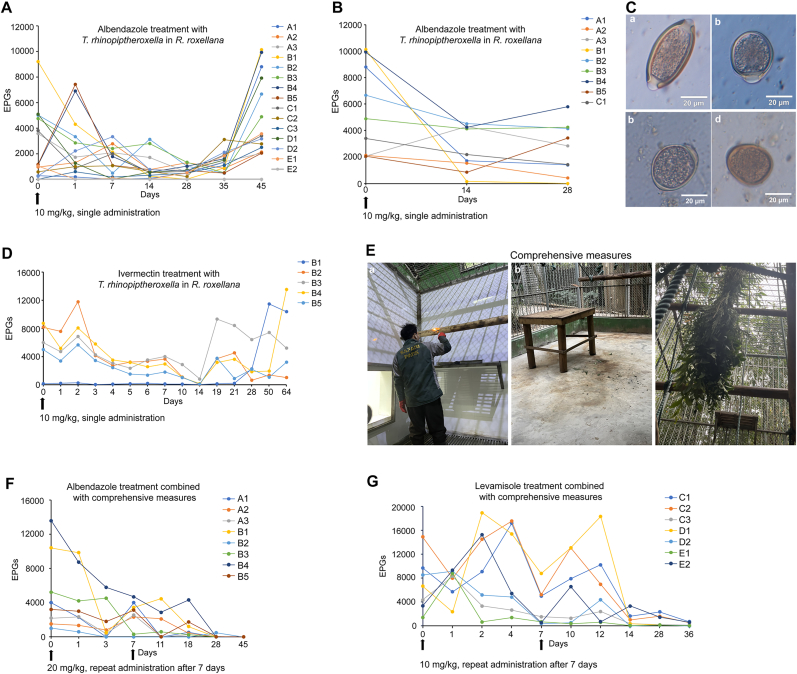
**Efficacy of anthelmintic administration and integrated strategies on *T. rhinopiptheroxella* in *R. roxellana*.** (A–B) The effects of albendazole administration on the dynamic changes of *T. rhinopiptheroxella* EPGs in *R. roxellana*. (C) Eggs morphology of *T. rhinopiptheroxella* with albendazole administration of the *R. roxellana* family; a: normal egg, b–d: teratoid eggs. (D) Dynamic changes of *T. rhinopiptheroxella* EPGs in *R. roxellana* after ivermectin administration. (E) Comprehensive measures to prevention and control of *T. rhinopiptheroxella* in *R. roxellana*. a: cage rinsing and flame deworming, b: converting sandy ground to concrete ground in the breeding region, c: food hanging from the ground. (F) Efficacy of the albendazole administration and improved categories to *T. rhinopiptheroxella* in *R. roxellana* living in the exhibition region. (G) Efficacy of levamisole administration of *T. rhinopiptheroxella* in *R. roxellana*. Arrow represented the administration time and the horizontal axis represented the days after administration.

**Fig. 4:**
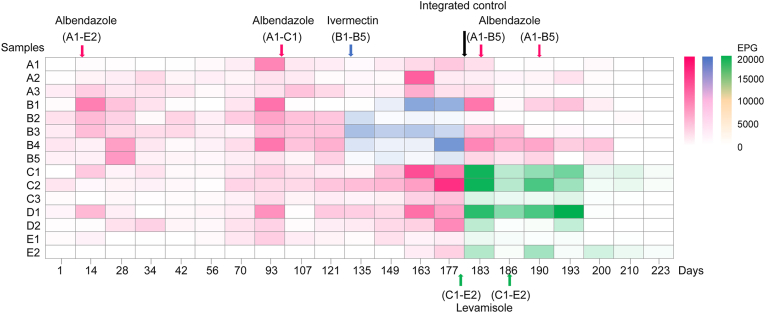
**Dynamic changes of *T. rhinopiptheroxella* EPGs in *R. roxellana* with anthelminthic treatment.** Pink means albendazole administration; blue means ivermectin administration; green means levamisole administration.

A fecal egg count reduction test was conducted to assess the efficacy of anthelmintic drugs against *T. rhinopiptheroxella* in *R. roxellana*. Three anthelmintic drugs (albendazole, levamisole and ivermectin) were employed to eliminate *T. rhinopiptheroxella* within the *R. roxellana* family (Table 2). First, 10 mg/kg of albendazole was administered orally to eliminate *T. rhinopiptheroxella* in 15 *R. roxellana*. The mean EPG exhibited a decline from 2455 to 2264 (a reduction of 7.8%) at 7 days post-antihelminthic treatment (dpa), to 1294 (a reduction of 47.3%) at 14 dpa and to 658 (a reduction of 73.2%) at 28 dpa. Additionally, 1 of 15 monkeys was negative for fecal eggs at 7 dpa. However, a notable increase in the EPG was observed after 35 dpa (Fig. 3, Fig. 4, and Table 2). Second, a further oral administration of 10 mg/kg albendazole was given to 9 monkeys in groups A1-C1. The mean EPG decreased from 5562 to 2626 (a reduction of 52.8%) at 14 dpa and to 2636 (a reduction of 52.6%) at 28 dpa. Only one monkey was negative for fecal eggs (Fig. 3, Fig. 4, and Table 2), indicating that the reduction rate of EPG was limited. Third, 10 mg/kg ivermectin was administered to eliminate *T. rhinopiptheroxella* in 5 monkeys belonging to group B. The mean EPGs decreased from 5610 to 2353 at 7 dpa (a reduction of 55.6%), and subsequently to 192 (a reduction of 96.6%). Additionally, 2 of 5 monkeys were negative for fecal eggs at 14 dpa. However, the EPGs increased to 2659 (a reduction of 52.6%) at 28 dpa (Fig. 3, Fig. 4, and Table 2). These results indicated that 10 mg/kg albendazole or ivermectin was initially effective, but the EPG gradually increased less than one month, indicating that the treatment was unable to effectively eliminate *T. rhinopiptheroxella* in *R. roxellana* (Fig. 3, Fig. 4, and Table 2).

**Table 2:** Analysis of fecal EPG in *R. roxellana* after anthelminthic drug administration.

Group (number)	Dosage	Physical measure	EPG before administration	EPG (reduction rate %)			Negative monkey		
				7	14	28	7	14	28
A, B, C, D, E (15)	Albendazole, 10 mg/kg, single administration	No	2455	2264 (7.8)	1294 (47.3)	658 (73.2)	1/15	0/15	1/15
A, B and C1 (9)	Albendazole, 10 mg/kg, single administration	No	5562	–	2626 (52.8)	2636 (52.6)	–	0/9	1/9
B (5)	Ivermectin, 10 mg/kg, single administration	No	5610	2353 (55.6)	192 (96.6)	2659 (52.6)	0/5	2/5	0/5
A, B (8)	Albendazole, 20 mg/kg, repeat administration after 7 days	Yes	5135	2549 (50.4)	1251 (75.6)	63 (98.8)	1/8	3/8	6/8
C, D, E (7)	Levamisole, 10 mg/kg, repeat administration after 7 days	Yes	7389	3176 (57.0)	983 (86.7)	869 (88.2)	0/7	1/7	0/7

The administration of albendazole resulted in the emergence of malformed eggs. The malformed eggs exhibited a length of 42.29 ± 6.66 μm and a width of 31.92 ± 3.99 μm, which were smaller than the size of a typical eggs (Fig. 3C). The eggs exhibited a subrounded shape and lacked one or two egg plugs, a feature that distinguished them from the barrel-shaped eggs with two egg plugs at both ends. The malformed eggs were unable to develop into infective eggs containing larvae under *in vitro* culture. Sequencing of ITS1 gene showed that the malformed eggs were identified as *T. rhinopiptheroxella* (Fig. S6). A 272 bp fragment of β-tubulin gene with three putative mutation sites (167, 198, and 200) for albendazole resistance (Mendes de Oliveira et al., 2022) was amplified from 14 fecal DNA samples by nested PCR (Fig. S7). No mutation was identified at the 167, 198, and 200 sites of β-tubulin gene. The reduction in fecal EPG and the emergence of malformed eggs following albendazole administration provided evidences that the worms were susceptible to albendazole.

### 3.6 Improved measures on the prevention and control of *T. rhinopiptheroxella* in the *R. roxellana* family

To improve the efficacy of the treatment for *T. rhinopiptheroxella* infection, a series of combined measures were implemented, including increasing dose, cage flushing and flame deworming, and hanging food from the floor (Fig. 3E). Results showed a significant reduction in the mean EPG of monkeys in groups A and B following administration of 20 mg/kg albendazole. The reduction was observed to be 50.4%, with the mean EPG decreasing from 5135 to 2549. Additionally, 1 of 8 monkeys was negative for fecal eggs at 7 dpa (Fig. 3, Fig. 4 and Table 2). The mean EPG of monkeys in groups A and B exhibited a further decline, reaching 1251 (a reduction of 75.6%), and 3 of 8 monkeys were negative for fecal eggs at 14 dpa (Fig. 3, Fig. 4 and Table 2). The mean EPG of monkeys in groups A and B exhibited a further reduction, reaching 63 (a reduction of 98.8%), and 6 of 8 monkeys were negative for fecal eggs at 28 dpa (Fig. 3, Fig. 4 and Table 2). The EPG of monkeys remained negative at 45 dpa. Therefore, a combined method resulted in a sustained reduction in EPG levels to a negative or low level in monkeys in groups A and B in the exhibition region.

Additionally, 10 mg/kg of levamisole was employed to eliminate worms in the monkeys in groups C, D, and E in the breeding region, while no other environmental or dietary treatments were administered within 7 dpa. The mean EPG of monkeys in groups C, D, and E decreased from 7389 to 3176 (a reduction of 57.0%) at 7 dpa. Only monkeys C3, D2, and E1 showed a consistent decline in EPG (Fig. 3, Fig. 4, and Table 2). Subsequently, levamisole was administered at 7 dpa in conjunction with integrated environmental and dietary interventions. The mean EPG of monkeys in groups C, D, and E continued to decline, reaching 983 (a reduction of 86.7%), and 1 of 7 monkeys was negative for fecal eggs at 14 dpa. The mean EPG continued to decline, reaching 869 (a reduction of 88.2%) at 28 dpa (Fig. 3, Fig. 4 and Table 2). Fecal examination showed that monkeys D1 and D2 were negative for eggs at 36 dpa. Therefore, the administration of 10 mg/kg levamisole in conjunction with combined measures was effective in maintaining a low EPG from 14 dpa. Additionally, the incidence of diarrhea among the *R. roxellana* family was markedly reduced from 31.7% to 2.7%, following the implementation of this combined treatment regimen (Table 3).

**Table 3:** Diarrhea rate of *R. roxellana* before and after treatment and improved strategies.

Administration	Means % (Range %)	T1 (<5%)	T2 (5%–15%)	T3 (15%–25%)	T4 (>25%)
Before	31.7 (10–60)	–	A2, D2, E1, E2 (4)	A1, B5, C2, C3 (4)	A3, B1, B2, B3, B4, C1, D1 (7)
After	2.7 (0–20)	A1-E2 (15)	–	–	–

## 4 Discussion

*R. roxellana* is one of the endemic and endangered species in China and is widely captive in wildlife zoos and reserve centers in China (Liu et al., 2024). *R. roxellana* is susceptible to *T. rhinopiptheroxella*, a soil-transmitted intestinal nematode. Considering the limited efficacy of current approach in removing *T. rhinopiptheroxella* in *R. roxellana*, we sought to develop an improved regimen to control *T. rhinopiptheroxella* in 15 captive *R. roxellana* at a wildlife zoo in southern China.

### 4.1 Identification of *T. rhinopiptheroxella* with host adaptation to *R. roxellana*

Microscopic examination and molecular detection showed that the observed worm in the *R. roxellana* family was *T. rhinopiptheroxella*, which has been previously identified in *R. roxellana* from Shaanxi Province, northwestern China (Wang et al., 2019). The observed eggs could develop into infective eggs following hatching *in vitro* culture, which is consistent with *Trichuris* species (Klementowicz et al., 2012). *T. rhinopiptheroxella* in the *R. roxellana* family was observed to be clustered with *Trichuris* species in non-human primates of *Trachypithecus francoisi* (François's langur) and *Papio hamadryas* (Hamadryas baboon), indicating that *T. rhinopiptheroxella* may be a non-zoonotic parasitic nematode. Phylogenetic analysis showed that this *T. rhinopiptheroxella* was clustered with *Trichuris* species from *R. roxellana* in different zoos in southern China into one branch, suggesting that *T. rhinopiptheroxella* might have been widely parasitized in captive *R. roxellana* in China, especially in southern China.

### 4.2 Limited effects of regular anthelmintic administration to *T. rhinopiptheroxella* infection

The administration of albendazole resulted in the formation of malformed eggs with a subround shape and the absence of one or two egg plugs in the feces of *R. roxellana*, which is consistent with teratogenic effects of albendazole as previous report (Chai et al., 2021). The malformed eggs were unable to develop into infective eggs containing larvae under *in vitro* culture. Moreover, no β-tubulin mutation site associated with albendazole resistance was identified in *T. rhinopiptheroxella*. Thus, *T. rhinopiptheroxella* in the *R. roxellana* family is sensitive to albendazole treatment, however, the administration of both albendazole and ivermectin resulted in only a transient reduction in EPG and was unable to eliminate *T. rhinopiptheroxella* from *R. roxellana*. A previous study of mebendazole administration also showed a reduction in the EPG of *R. roxellana* at 7 dpa, however, the EPG increased to a high level at 50 dpa (He et al., 2017). Therefore, anthelmintics had a restricted effect on *R. roxellana* with *T. rhinopiptheroxella* infection.

As for the reasons, a comprehensive analysis was conducted on drug dosage, environmental factors, behavioral patterns, and feeding habits of *R. roxellana*. Firstly, the standard dosage of oral albendazole is 20 mg/kg of body weight, with a repeat dose administered one week later, or at a dose of 400 mg per adult animal for three to five consecutive days according to the medication guide. However, the dosage of anthelmintics utilized in this study was 10 mg/kg, administered in a single dose. Secondly, the activity areas were characterized by sandy soil in the breeding region and a semi-forested environment in the exhibition region, which presented a significant challenge in the removal of eggs from the soils within the activity area. Thirdly, the eggs might be deposited on the skin or fingertips of *R. roxellana*, and were subsequently transmitted from hand to mouth through the act of intimate grooming. Thus, it can be surmised that the fecal-oral transmission of *T. rhinopiptheroxella* eggs could easily result in repeated infection. Furthermore, the presence of leaves on the tables in the activity area provided an additional source of contamination, as the monkeys had free access to these foods and could potentially ingest the eggs present on them. Therefore, the presence of these eggs in the soil and on contaminated foodstuffs ultimately resulted in the occurrence of infection.

### 4.3 Improved categories for anthelmintic treatment to *T. rhinopiptheroxella* infection

The implementation of comprehensive management strategies, encompassing dosage, environmental conditions, cage, floor, and food, has the potential to eliminate eggs in the environment and improve environmental contamination, thereby increasing the efficacy of anthelmintic drugs. These comprehensive measures were crucial for controlling reinfection with *T. rhinopiptheroxella* in *R. roxellana*. Furthermore, correlation analysis showed a significant correlation between the EPG level and diarrhea in *R. roxellana*. The combined regimen was observed to reduce the incidence of diarrhea in the monkeys to a minimal level, suggesting that *T. rhinopiptheroxella* infection was the causative agent to the diarrhea of *R. roxellana*. Thus, it can be concluded that infected *R. roxellana* was the source of transmission of *T. rhinopiptheroxella,* consequently, controlling the reinfection of *T. rhinopiptheroxella* is the key to the treatment of trichuriasis in *R. roxellana*.

## 5 Conclusion

A comprehensive analysis revealed that *T. rhinopiptheroxella* was prevalent in captive *R. roxellana*. Moreover, anthelmintic treatment alone reduced the EPG level in the short term, but could not eliminate *T. rhinopiptheroxella* in *R. roxellana*. A combined treatment regimen comprising appropriate anthelmintic doses, and environmental and dietary managements has been demonstrated to be efficacious in reducing the EPG to negative or very low levels. This study provides evidence for the control of *Trichuris* infection in captive *R. roxellana*.

## CRediT authorship contribution statement

**Zhengjiu Ren:** Writing – original draft, Methodology, Investigation, Conceptualization. **Jinzhi Lu:** Methodology, Investigation. **Tianyou Zhang:** Resources, Investigation. **Lihua Xiao:** Writing – review & editing, Methodology, Funding acquisition, Conceptualization. **Peng Zhang:** Writing – review & editing, Resources, Investigation. **Guixin Dong:** Resources, Investigation. **Yaoyu Feng:** Writing – review & editing, Methodology, Conceptualization. **Dongjuan Yuan:** Writing – review & editing, Writing – original draft, Methodology, Investigation, Funding acquisition, Conceptualization.

## Ethics statement

All fecal samples utilized in this study were obtained from *R. roxellana* with the approval of the zoo. All experimental procedures were conducted in accordance with the Animal Ethical Procedures and Guidelines of the People's Republic of China. The research protocol was reviewed and approved by the Ethics Committee of South China Agricultural University, People's Republic of China.

## Availability of data and materials

Nucleotide sequence data reported in this paper are available in GenBank under accession numbers PQ247221 (mitogenome of *T. rhinopiptheroxella*) and OR563921 (ITS1 sequence of *T. rhinopiptheroxella*).

## Funding

This work was supported by Guangdong Chimelong Philanthropic Foundation (CLPF2021007Z), the 10.13039/501100001809National Natural Science Foundation of China (32473055), 10.13039/501100021171Guangdong Basic and Applied Basic Research Foundation (2023A1515011964 and 2024A1515011658), Double First-class Discipline Promotion Project (2023B10564003).

## Declaration of competing interest

The authors declare that they have no known competing financial interests or personal relationships that could have appeared to influence the work reported in this paper.
