# Supplementary material for: A combined treatment regimen for Trichuris rhinopiptheroxella infection in Rhinopithecus roxellana in southern China
Source: Int J Parasitol Parasites Wildl. 2025 Jan 3;26:101036. doi: 10.1016/j.ijppaw.2025.101036 (PMC11762197; doi:10.1016/j.ijppaw.2025.101036)
Supplement: Multimedia component 1 [file mmc1.docx]

**Supplementary Files:**


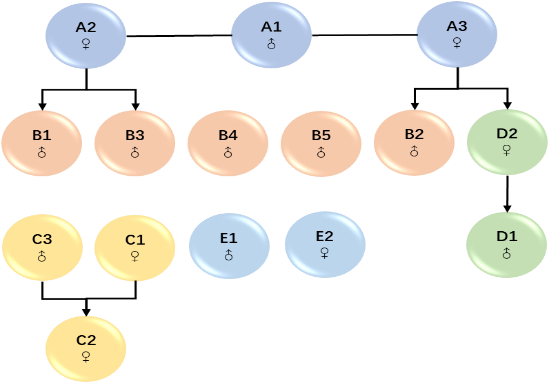


**Figure S1 The relationship and the groups of 15 *R. roxellana* in a wildlife zoo of the southern China.** The circle with the same color means the *R. roxellana* in the same group; The arrow indicates the offspring.


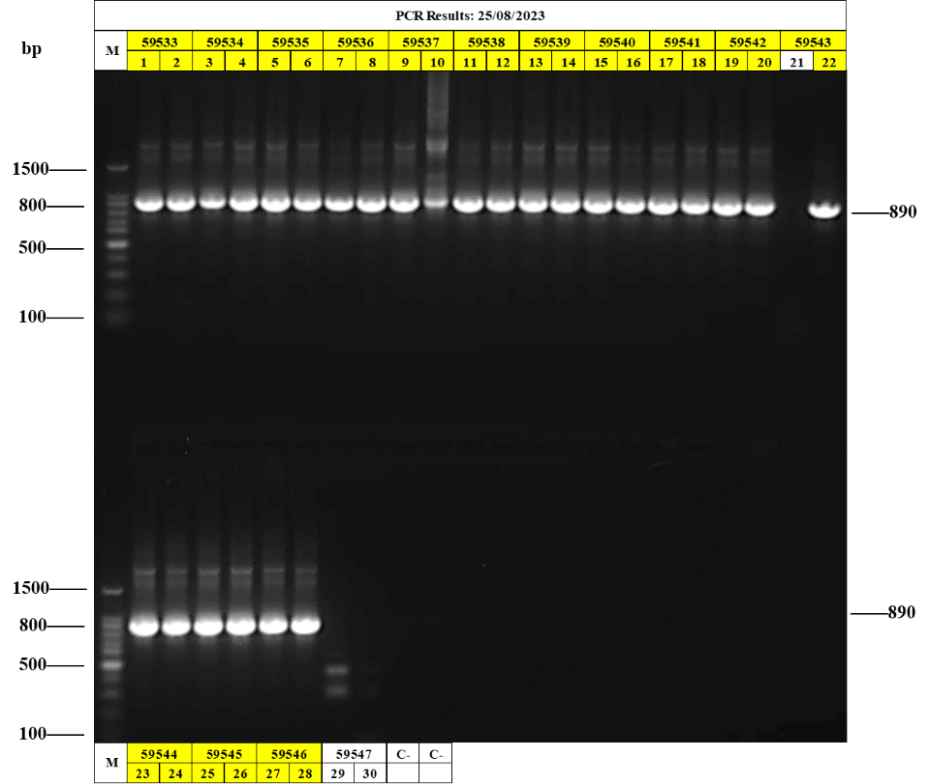


**Figure S2 PCR amplification of ITS1 gene from *Trichuris* eggs in the fecal sample of *R. roxellana.*** Each fecal sample from *R. roxellana* performed repeated PCR reactions. M: marker，C- mean negative control.


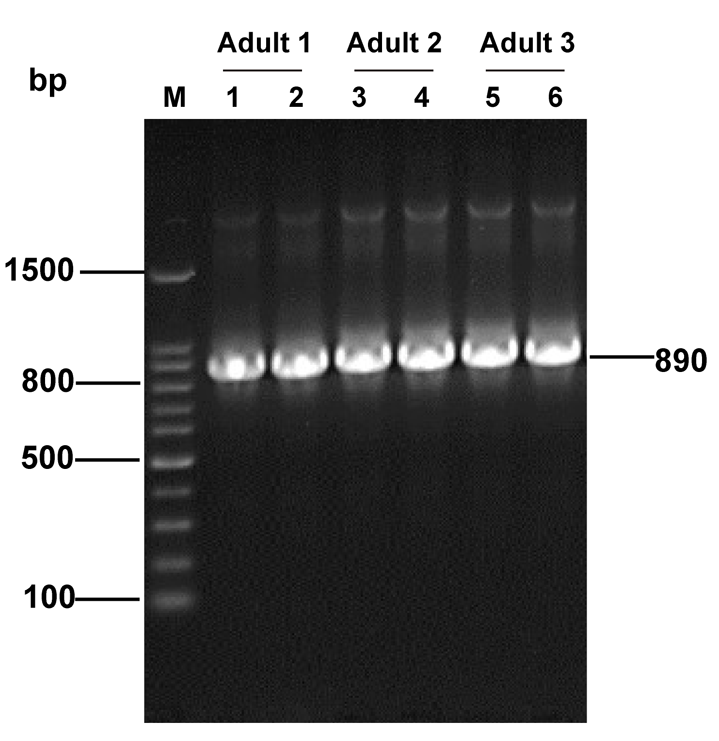


**Figure S3 PCR amplification of ITS1 gene of *Trichuris* adult worms from the fecal sample of *R. roxellana.*** M: marker.


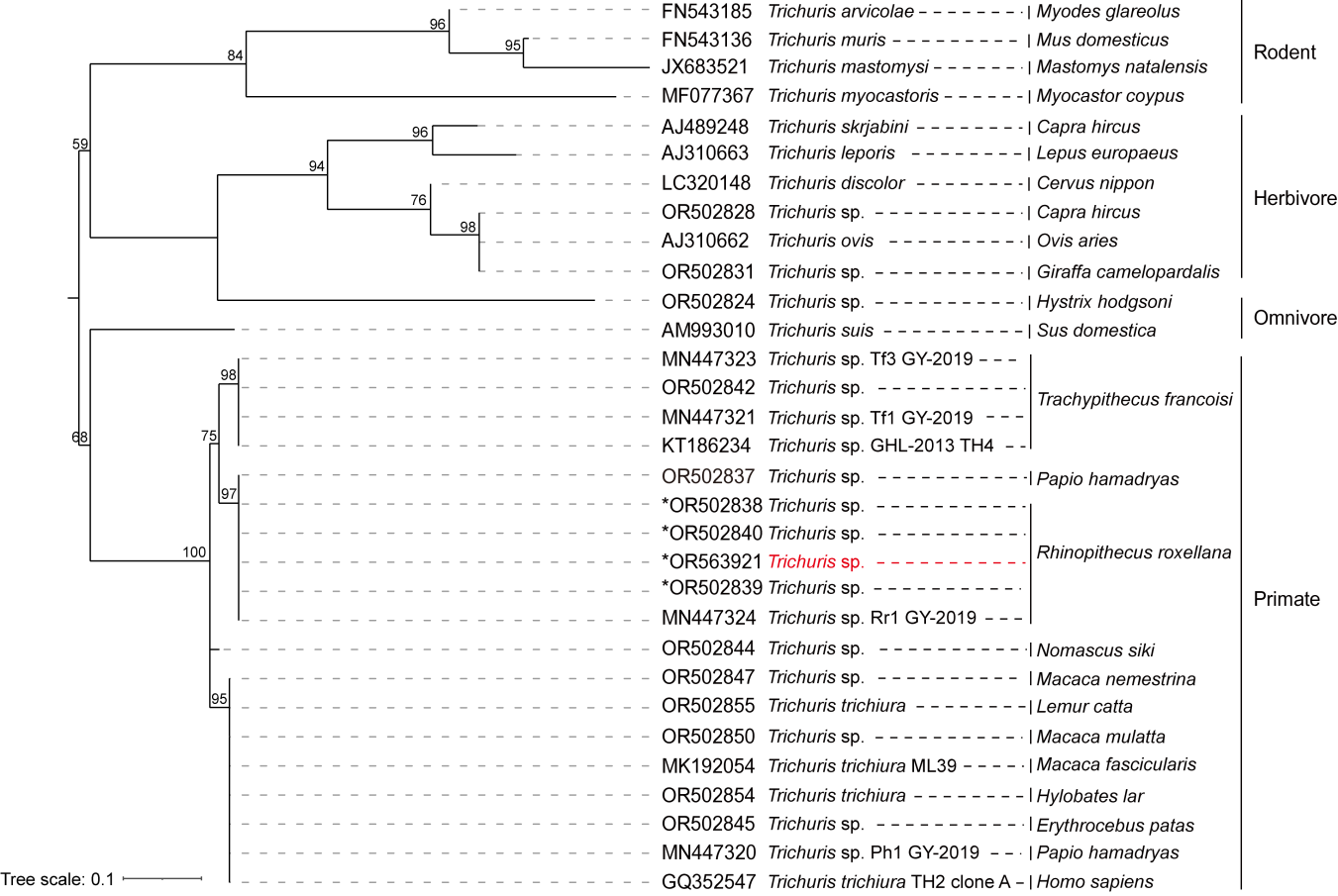


**Figure S4 Phylogenetic relationship of *Trichuris* species from animals based on a maximum-likelihood analysis of ITS1 sequences.** Bootstrap values were generated using 1000 replicate analyses. Nodes with bootstrap support values of 50 or greater were indicated. ITS1 sequence *Trichuris* sp*.* from *R. roxellana* were indicated by red. * means *Trichuris* species in *R. roxellana* from different zoos in southern China (Li et al., 2024).

**
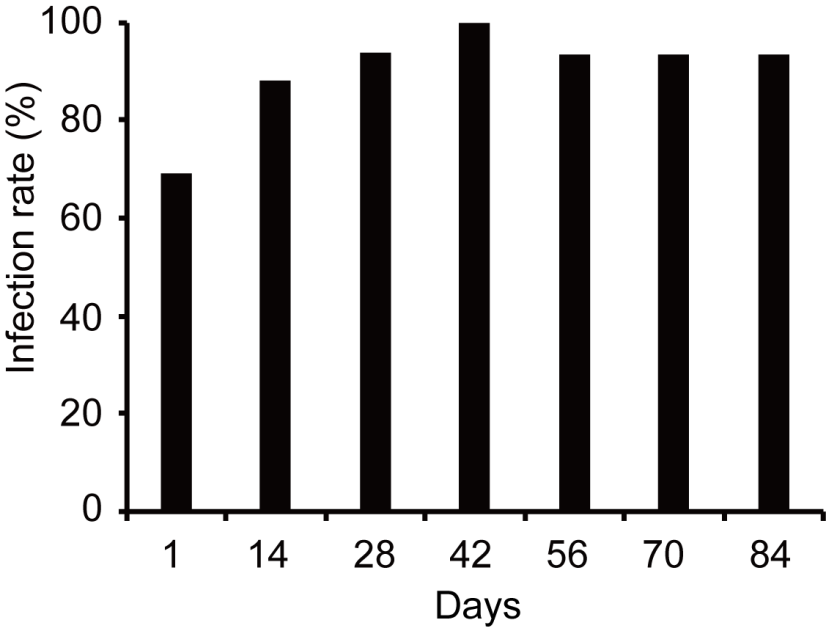
**

**Figure S5 Infection rate of** ***T. rhinopiptheroxella* in *R. roxellana* during approximately three months investigation*.***


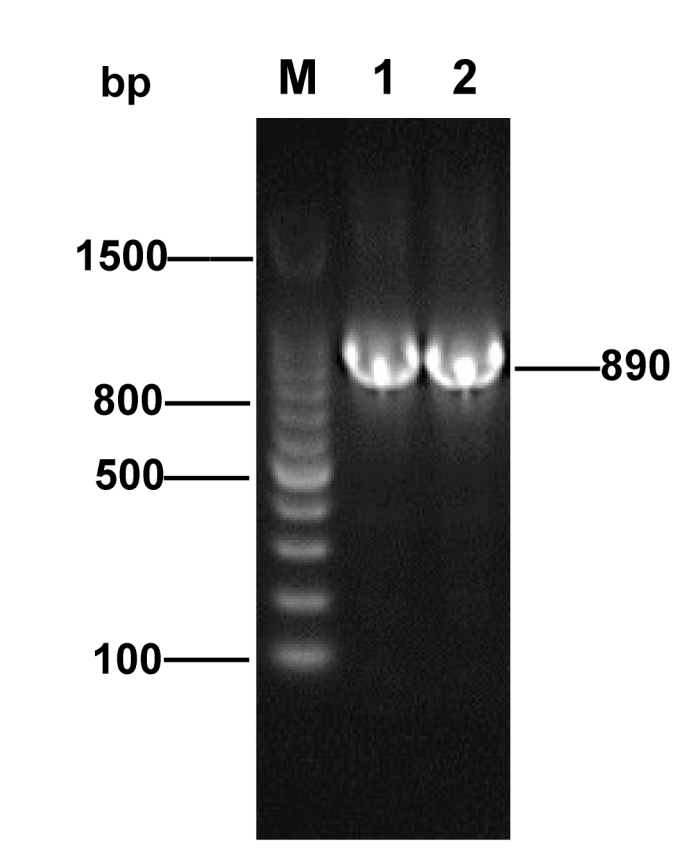


**Figure S6 PCR amplification of ITS1 gene from the malformed eggs of *T. rhinopiptheroxella.***


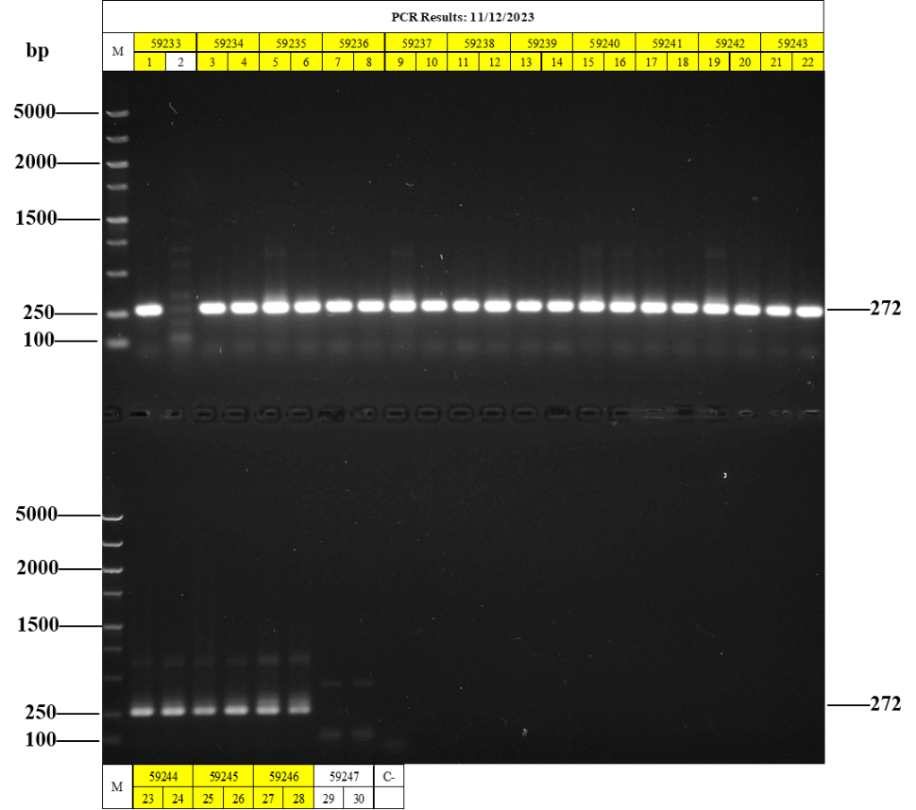


**Figure S7 PCR amplification of *β-tubulin* gene of *T. rhinopiptheroxella* from *R. roxellana.*** Each fecal sample from *R. roxellana* performed repeated PCR reactions. M: marker，C- mean negative control.

**Table S1 Primers for PCR amplification of *Trichuris* species ITS1**

| Locus | Primer sequences (5’→3’) | Annealing temperature (℃) | Sequence length (bp) |
| --- | --- | --- | --- |
| ITS1 | ITS1_1417F：AGGGACCAGCGACACTTTC  ITS1_2505R：GAGTGTCACGTCGTTCTTCAAC  ITS1_1567F：GTTCTCGTGACTGGGAC  ITS1_2462R：CTACGAGCCAAGTGATCC | 50  50 | ~1080  ~890 |

**Table S2** **Mitogenome organization, start and stop codons of PCGs, and anticodons of tRNA of the worm from *R. roxellana***

| Gene | Start-end | | | Length | | | | | | | | Start/stop codon | | | | Anticodon | | | |  |  |  |
| --- | --- | --- | --- | --- | --- | --- | --- | --- | --- | --- | --- | --- | --- | --- | --- | --- | --- | --- | --- | --- | --- | --- |
|  |  |  |  | No nt | | | | No a.a | | | |  |  |  |  |  |  |  |  |  |  |  |
| *cox*1 | 2-1546 | | | 1545 | | | | 514 | | | | ATG/TAA | | | |  | | | |  |  |  |
| *cox*2 | 1553-2227 | | | 675 | | | | 224 | | | | ATG/TAA | | | |  | | | |  |  |  |
| *trn*L2 | 2240-2304 | | | 65 | | | |  | | | |  | | | | TAA | | | |  |  |  |
| *trn*E | 2317-2374 | | | 58 | | | |  | | | |  | | | | TTC | | | |  |  |  |
| *nad*1 | 2390-3289 | | | 900 | | | | 299 | | | | ATA/TAG | | | |  | | | |  |  |  |
| *trn*K | 3387-3450 | | | 64 | | | |  | | | |  | | | | TTT | | | |  |  |  |
| *nad*2 | 3455-4354 | | | 900 | | | | 299 | | | | ATA/TAA | | | |  | | | |  |  |  |
| *trn*M | 4355-4415 | | | 61 | | | |  | | | |  | | | | CAT | | | |  |  |  |
| *trn*F | 4416-4472 | | | 57 | | | |  | | | |  | | | | GAA | | | |  |  |  |
| *nad*5 | 4478-6025 | | | 1548 | | | | 515 | | | | ATA/TAA | | | |  | | | |  |  |  |
| *trn*H | 6023-6077 | | | 55 | | | |  | | | |  | | | | GTG | | | |  |  |  |
| *trn*R | 6079-6139 | | | 61 | | | |  | | | |  | | | | TCG | | | |  |  |  |
| *nad*4 | 6141-7361 | | | 1221 | | | | 406 | | | | ATT/TAA | | | |  | | | |  |  |  |
| *nad*4L | 7388-7621 | | | 234 | | | | 77 | | | | ATA/TAA | | | |  | | | |  |  |  |
| *trn*T | 7642-7697 | | | | 56 | | | |  | | | |  | | | | TGT | | | |  |  |
| *trn*P | 7698-7749 | | | | 52 | | | |  | | | |  | | | | TGG | | | |  |  |
| *nad*6 | | 7743-8219 | | | | 477 | | | | 158 | | | | ATT/TAA | | | |  | | | |  |
| *cyt*b | | 8234-9340 | | | | 1107 | | | | 368 | | | | ATG/TAG | | | |  | | | |  |
| *trn*S1 | | 9339-9389 | | | | 51 | | | |  | | | |  | | | | TCT | | | |  |
| *rrn*S | | 9402-10091 | | | | 690 | | | |  | | | |  | | | |  | | | |  |
| *trn*V | | 10091-10147 | | | | 57 | | | |  | | | |  | | | | TAC | | | |  |
| *rrn*L | | 10147-11147 | | | | 1001 | | | |  | | | |  | | | |  | | | |  |
| *atp*6 | | 11115-11953 | | | | 839 | | | | 279 | | | | ATA/TA | | | |  | | | |  |
| *cox*3 | | | 11942-12715 | | | | 774 | | | | 257 | | | | ATG/TAA | | | |  | | | |
| *trn*W | | | 12720-12787 | | | | 68 | | | |  | | | |  | | | | TCA | | | |
| *trn*Q | | | 12798-12853 | | | | 56 | | | |  | | | |  | | | | TTG | | | |
| *trn*I | | | 12857-12920 | | | | 64 | | | |  | | | |  | | | | GAT | | | |
| *trn*G | | | 12924-12980 | | | | 57 | | | |  | | | |  | | | | TCC | | | |
| *tnr*D | | | 12987-13042 | | | | 56 | | | |  | | | |  | | | | GTC | | | |
| *atp*8 | | | 13043-13192 | | | | 150 | | | | 49 | | | | ATT/TAG | | | |  | | | |
| *nad*3 | | | 13203-13544 | | | | 342 | | | | 113 | | | | ATT/TAA | | | |  | | | |
| *trn*S2 | | | 13649-13701 | | | | 53 | | | |  | | | |  | | | | GAC | | | |
| *trn*N | | | 13701-13757 | | | | 57 | | | |  | | | |  | | | | GTT | | | |
| *trn*L1 | | | 13768-13832 | | | | 65 | | | |  | | | |  | | | | TAG | | | |
| *trn*A | | | 13838-13892 | | | | 55 | | | |  | | | |  | | | | GAC | | | |
| *trn*C | | | 13936-13992 | | | | 57 | | | |  | | | |  | | | | GCA | | | |
| *trn*Y | | | 14000-14058 | | | | 59 | | | |  | | | |  | | | | GTA | | | |

**Table S3 Multifactorial nonparametric analysis of EPG in *R. roxellana***

|  | Median (P25, P75) | | | | | |
| --- | --- | --- | --- | --- | --- | --- |
|  | Gender | Age | Co-inhabitation | Living region | Inter -subgroups | Diarrhea grade |
| EPG | 1467 (912, 2984) | | | | | |
| *H/*Z value | -0.857 | 4.156 | 14.0 | -0.347 | 7.282 | 7.591 |
| *P* | 0.391 | 0.245 | 0.45 | 0.728 | 0.122 | 0.022* |

Note: *: *P* < 0.05；*P* > 0.05 means no significant.

**Table S4 The association between diarrhea and exposure factors of *R. roxellana***

| Risk factor | OR（95% CI） | *P* |
| --- | --- | --- |
| EPG | 1.002（1.001~1.002） | <0.01*** |

OR: Odd ratio；CI: confidence interval.

**Reference:**

Li, H., Ren, Z., Wang, W., Shen, F., Huang, J., Wang, C., Lu, J., Pan, X., Xiao, L., Feng, Y., Yuan, D., 2024. A combined amplicon approach to nematode polyparasitism occurring in captive wild animals in southern China. Parasit Vectors 17, 94.
